# *In vivo* antimalarial activity of crude extracts and solvent fractions of leaves of *Strychnos mitis* in *Plasmodium berghei* infected mice

**DOI:** 10.1186/s12906-016-1529-7

**Published:** 2017-01-05

**Authors:** Selamawit Fentahun, Eyasu Makonnen, Tesfaye Awas, Mirutse Giday

**Affiliations:** 1 Wollo University, P.O. Box 1145, Dessie, Ethiopia; 2 School of Medicine, Addis Ababa University, P.O. Box 1176, Addis Ababa, Ethiopia; 3 Institute of Biodiversity, P.O. Box 30726, Addis Ababa, Ethiopia; 4 Aklilu Lemma Institute of Pathobiology, Addis Ababa University, P.O. Box 1176, Addis Ababa, Ethiopia

**Keywords:** *Strychnos mitis*, Crude extract, Fraction, *Plasmodium berghei*, Antimalarial activity, Ethiopia

## Abstract

**Background:**

Malaria is a major public health problem in the world which is responsible for death of millions particularly in sub-Saharan Africa. Today, the control of malaria has become gradually more complex due to the spread of drug-resistant parasites. Medicinal plants are the unquestionable source of effective antimalarials. The present study aimed to evaluate antiplasmodial activity and acute toxicity of the plant *Strychnos mitis* in *Plasmodium berghei* infected mice.

**Methods:**

Standard procedures were employed to investigate acute toxicity and 4-day suppressive effect of crude aqueous and hydro-methanolic extracts of the leaves of *Strychnos mitis* against *P. berghei* in Swiss albino mice. Water, n-hexane and chloroform fractions, obtained from crude hydro-methanolic extract, were also tested for their suppressive effect against *P. berghei*.

**Results:**

All crude extracts revealed no obvious acute toxicity in mice up to the highest dose administered (2000 mg/kg). All crude and solvent fractions of the leaves of *Strychnos mitis* inhibited parasitaemia significantly (*p* < 0.01). At the highest dose of 600 mg/kg, both aqueous and hydro-methanolic extracts demonstrated higher performance with 95.5 and 93.97% parasitaemia suppression, respectively. All doses of crude extracts and fractions of leaves of *Strychnos mitis* prolonged survival time of infected mice dose dependently. The highest two doses of the crude aqueous and hydro-methanolic extracts, and chloroform and aqueous fractions prevented weight loss in a dose dependent manner. Whereas, all doses of n-hexane fraction prevented loss of body weight but not in a dose dependent manner. The crude aqueous extract at the doses of 400 mg/kg and 600 mg/kg and hydro-methanolic extract at all dose levels significantly (*p* < 0.01) prevented packed cell volume reduction. Crude aqueous extract at a dose of 600 mg/kg and hydro-methanolic extract at all dose levels significantly prevented temperature reduction. Phytochemical screening of the crude aqueous and hydro-methanolic extracts revealed the presence of alkaloids, anthraquinones, glycosides, terpenoids, saponins, tannins and phenols.

**Conclusion:**

The results of this study provide support the traditional therapeutic use of *Strychnos mitis* for treatment of malaria. However, further in-depth study is needed to evaluate the potential of the plant towards the development of new antimalarial agent.

## Background

Malaria still remains to be a critical problem to global public health. It continues to remain among the top three infectious diseases (Malaria, tuberculosis and HIV) affecting billions of people globally [1]. Malaria kills more than one million individuals in the tropical and subtropical zones annually [2]. Pregnant women and children under 5 years of age are the most vulnerable to the disease [3]. In Ethiopia, malaria affects four to five million people annually [4] resulting in 70,000 deaths [5]. Since the year 2000, malaria mortality rates have decreased worldwide and in Africa by 47 and 54%, respectively [6]. However, malaria control program has been jeopardized by lack of access to effective malaria control tools, emergence of resistance to antimalarial drugs and insecticides [7]. This calls for more effort to develop new antimalarial compounds with novel mechanisms of action. In recent times, natural products of plant sources have been the centre of focus as the main source of new, safer and more effective bioactive compounds with medicinal properties [8]. Medicinal plants have been the focus for search of new antimalarial drugs in various parts of the world. Artemisinin and quinine are drugs that have been developed from the herbaceous plants *Artemisia annua* L. and bark of *Cinchona pubescens* Vahl., respectively, based on ethnobotanical leads [9]. Such discoveries have inspired many researchers to look for new antimalarial drugs from plants.

In Ethiopia, some traditionally used antimalarial plants have been screened for their antiplasmodial activity. These include *Dodonaea viscosa* subsp. *angustifolia* (L.f.) J.G. West, *Clerodendrum myricoides* (Hochst.) Vatke, *Aloe debrana* Christian, *Adhathoda schimperiana* Hochst. ex Nees and *Asparagus africanus* Lam. Extracts of seeds of *Dodonaea viscosa* subsp. *angustifolia* that were tested against *Plasmodium berghei* in mice model significantly reduced parasitaemia and prevented packed cell volume reduction [10]. A study conducted by Deressa et al. [11] revealed strong activities of crude extracts of *Clerodendrum myricoides* and *Aloe debrana* against *P. berghei.* A study by Petros & Melaku [12] reported significant parasitaemia reduction by hydro-alcoholic extract of leaves of *A. schimperiana* tested against chloroquine-sensitive *P. berghei*. Dikasso et al. [13] also reported that hydro-alcoholic extracts of *Asparagus africanus* demonstrated appreciable in vivo antimalarial activity against *P. berghei.*

A report shows that the plant *Strychnos mitis* S.Moore (Loganiaceae) is traditionally used in Asia to treat malaria [14]. Some in vitro and in vivo studies indicate the antmalarial activity of extracts from *Strychnos* species. An in vitro study revealed a very promising activity by methanolic extract of *Strychnos variabilis* De Wild. and interesting activity by that of *Strychnos mellodora* S.Moore and *Strychnos* g*ossweileri* Excell, all close relatives of *Strychnos mitis* [15]. An in vitro study conducted on several alkaloids extracted from *Strychnos* species showed high and selective activity of quasidimetric alkaloids against *Plasmodium falciparum* [16]. Another *in vitro* study demonstrated high activity of some compounds extracted from *Strychnos icaja* Baill. [17]. *Strychnos spinosa* Lam. [18] and *Strychnos usambarensis* Gilg ex Engl. [19] have been reported to have antiplasmodial activity in vitro. *Strychnos icaja* was reported to show potent antimalarial activity in vivo [20]. A study by Sanmugapriya and Venkataraman [21] revealed the antipyretic effect of the seeds of *Strychnos potatorum*, L.f. on experimental rats. However, the there is no report indicating evaluation of *Strychnos mitis* for its antiplasmodial activity. Thus, the aim of this study was to evaluate the in vivo antiplasmodial activity of the crude extracts and solvent fractions of the leaves of *Strychnos mitis* in mice infected with chloroquine sensitive *P. berghei*.

## Methods

### Plant sample collection

For the in vivo test, plant samples of *Strychnos mitis* were collected in February 2014 from around Yirgalem town, South Region of Ethiopia, located at 318 km south of Addis Ababa. Voucher specimen (SF-001) of the plant was also collected, identified and deposited at the National Herbarium of the Addis Ababa University (AAU) for future referencing.

### Preparation of crude extracts

Leaf samples of the plant were air-dried at room temperature under shade in the preparation room of the Aklilu Lemma Institute of Pathobiology (ALIPB), AAU. The dried leaves were ground to powder using mortar and pestle. Crude extracts were prepared by cold maceration techniques as outlined by O’Neill et al. [22]. Leaf powders (300 g each) were soaked in 2400 ml of 80% methanol and 2700 ml of distilled water in separate Erlenmeyer flasks. The flasks containing the plant powders dissolved in methanol and distilled water were placed on orbital shaker (Thermoforma, USA) of 145 rotations per minute (rpm) for 72 and 24 h, respectively. The mixtures were filtered using gauze and filtrates were passed through Whatman filter paper number 1 with pore size of 150 mm diameter (Wagtech international Ltd, England). The residues were re-macerated twice. The methanol in the filtrate of the hydro-methanolic extract was removed under reduced pressure by rotary evaporator (Buchi type TRE121, Switzerland) at 45 rpm and 40 °C to obtain crude extract. The extract was further concentrated to dryness with a lyophilizer (Wagtech Jouan Nordic DK-3450 Allerod, Denmark) at −50 °C and vacuum pressure (200 mBar). The aqueous extract was frozen in deep freezer overnight and then freeze dried with a lyophilizer (Wagtech Jouan Nordic DK-3450 Allerod, Denmark) at −50 °C and vacuum pressure (200 mBar). All extracts were stored in screw cap vials in a refrigerator (AKIRA, China) at −4 °C until use. The water extract was dissolved in distilled water, and the 80% methanol extract in 2% Tween 80 before oral administration.

### Preparation of fraction of hydro-methanolic crude extracts

The crude hydro-methanolic extract was subjected to fractionation using n-hexane and chloroform. Forty gram of the extract was suspended in a separatory funnel in 240 ml of distilled water and partitioned with 3 × 240 ml n-hexane. The filtrate was concentrated in a rotary evaporator (Buchi type TRE121, Switzerland) at 45 rpm and 40 °C to obtain the n-hexane fraction. The aqueous residue was then partitioned with 3 × 240 ml chloroform. The chloroform filtrate was concentrated to obtain chloroform fraction using same method used to get n-hexane fraction. The remaining aqueous residue was frozen in deep freezer overnight and then freeze dried with a lyophilizer (Wagtech Jouan Nordic DK-3450 Allerod, Denmark) at −50 °C and vacuum pressure (200 mBar) to obtain aqueous fraction. All fractions were stored in screw cap vials in a refrigerator (AKIRA, China) at −4 °C until use. The n-hexane and chloroform fractions were separately dissolved in 3% Tween 80 and aqueous fraction was dissolved in distilled water before oral administration.

### Phytochemical screening

The 80% methanol and aqueous extracts of leaves of *Strychnos mitis* were screened for the presence of secondary metabolites to relate the antimalarial activity of the plant with the presence or absence of these constituents. Thus, tests for alkaloids, saponins, cardiac glycosides, flavonoids, terpenoids, steroids, phenols and tannins were performed using standard procedures [23, 24].

### Experimental animals and parasite inoculation

Swiss albino mice, 6–8weeks of age and weighing 27–32 g, obtained from the Ethiopian Public Health Institute (EPHI), were used for the tests. Female mice were used for in vivo acute toxicity test and male mice were used for in vivo antimalarial screening. The mice were maintained in the animal house of ALIPB, AAU, under standard condition at room temperature by exposing them to 12 h light and 12 h dark cycle, with food and water *ad libitum*. Mice were handled based on internationally accepted guideline [25].

Chloroquine sensitive *P. berghei* (ANKA strain) was obtained from the Ethiopian public Health Institute (EPHI). The parasites were maintained by serial passage of blood from infected mice to non-infected ones on weekly basis [26]. Donor mice infected with a rising parasitaemia of 20–30% were used to infect mice in the 4-day suppressive test. The donor mice were sacrificed and blood was pooled together in a petri-dish containing 2% trisodium citrate (BDH chemicals, England) as anticoagulant to avoid variability in parasitaemia. The blood was then diluted with 0.9% normal saline so that each 0.2 ml of blood contained 1x10^7^ *P. berghei* infected erythrocytes. Each mouse used in the experiment was then inoculated intraperitoneally with 0.2 ml of the diluted blood.

### *In vivo* acute toxicity test

Crude aqueous and 80% methanol extracts of *S. mitis* were evaluated for their acute toxicity in non-infected female Swiss albino mice of 6–8 weeks old and weighing 27–32 g according to OECD Guideline No. 425 [27]. The mice were fasted overnight and weighted before test. A single female mouse was given 2000 mg/kg of the extract as a single dose by oral gavage. After administration of the extract, food was withheld for further 2 hours period. Death was not observed in the first 24 h. Then, additional four mice were given the same dose of the extract (2000 mg/kg). The mice were then observed for toxic signs in the next 14 days.

### *In vivo* antimalarial screening

In vivo antiplasmodial activity evaluation of the crude extracts (hydro-methanolic and aqueous extract) and three fractions (n-hexane, chloroform and aqueous fraction) of the leaves of *S. mitis* was carried out against *P. berghei* according to method described by Peters et al. [28] by randomly assigning 30 male mice into five groups (three treatment groups and two control groups). The three treatment groups received 200 mg/kg, 400 mg/kg and 600 mg/kg of the crude extracts and 100 mg/kg, 200 mg/kg and 400 mg/kg of the fractions, respectively, once daily for 4 days. The two controls (negative and positive) for crude extract and fractions received the vehicle (distilled water) and chloroquine phosphate (25 mg/kg) (standard drug), respectively. The vehicle, the plant extracts and the standard drug were administered orally (by oral gavage). The dose levels of the extracts and fractions were determined based on result obtained from oral acute toxicity test.

Treatment was started 3 hours after mice had been inoculated intraperitoneally with 0.2 ml of infected blood containing about 1×10^7^ parasites at day 0 by using a hypodermic needle [29] and then continued for additional 3 days (from day 1 to day 3). On the 5^th^ day (day 4), thin films were made from the tail blood of each mouse and smeared onto a microscope slide to make a film [30]. The blood films were fixed with methanol, stained with 10% Giemsa at pH 7.2 for 15 min and parasitaemia was examined microscopically to determine parasitaemia level and percentage parasite suppression. Moreover, each mouse was observed daily for determination of survival time.

### Determination of body weight and temperature

The body weight of each mouse in all the groups was taken before infection (day 0) and on day 4 using a sensitive weighing balance (METTLER TOLEDO, Switzerland). The rectal temperature of the mice was measured with a digital thermometer before infection and then daily up to day 4 to see the effect of the extracts and fractions on body temperature.

### Determination of packed cell volume

Packed cell volume (PCV) was measured to predict the effectiveness of the test extracts and fractions in preventing hemolysis resulting from increasing parasitaemia associated with malaria. Blood was collected from tail of each mouse in heparinized microhaematocrit capillary tubes. The capillary tubes were filled with blood up to ¾^th^ of their volume and sealed.

The tubes were sealed by crystal seal and placed in a microhematocrit centrifuge (Hettichhaematokrit, Germany) with sealed ends outwards and centrifugedfor 5 min at 11,000 rpm. PCV is a measure of the proportion of RBCs to plasma and measured before inoculating the parasite (day0) and after treatment (day4) [13] using the following relationship [10].

$$ \boldsymbol{P}\boldsymbol{C}\boldsymbol{V}=\frac{\boldsymbol{Volume}\ \boldsymbol{of}\ \boldsymbol{erythrocyte}\ \boldsymbol{in}\ \boldsymbol{a}\ \boldsymbol{given}\ \boldsymbol{volume}\ \boldsymbol{of}\ \boldsymbol{blood}}{\boldsymbol{Total}\ \boldsymbol{blood}\ \boldsymbol{volume}\ \boldsymbol{examined}} $$

### Determination of parasitaemia

On day 4 of the experiment, thin smears were prepared from tail blood on microscopic slides, dried and fixed with methanol. The blood films were stained with Giemsa and examined under the microscope. Five different fields on each slide were examined and the average was taken and percentage parasitaemia was determined using the formula described by Fidock et al. [26].

$$ \%\ \boldsymbol{Parasitaemia} = \frac{\boldsymbol{Number}\ \boldsymbol{of}\ \boldsymbol{infected}\ \boldsymbol{RBCs}}{\boldsymbol{Total}\ \boldsymbol{number}\ \boldsymbol{of}\ \boldsymbol{RBCs}\ \boldsymbol{examined}}\times \boldsymbol{100} $$

The percentage suppression of parasitaemia was calculated for each test concentration by comparing the parasitaemia in infected controls with those received different concentrations of the test extract.

$$ \%\ \boldsymbol{Suppression} = \frac{\boldsymbol{Parasitaemia}\ \boldsymbol{in}\ \boldsymbol{negative}\ \boldsymbol{control}\ \boldsymbol{\hbox{-}}\;\boldsymbol{parasitaemia}\ \boldsymbol{in}\ \boldsymbol{test}\ \boldsymbol{group}}{\boldsymbol{Parasitaemia}\ \boldsymbol{in}\ \boldsymbol{negative}\ \boldsymbol{control}}\times \boldsymbol{100} $$

### Determination of mean survival time

Mortality was monitored daily and the number of days from the time of inoculation of the parasite up to death was recorded for each mouse in the treatment and control groups throughout the follow up period. The mean survival time (MST) for each group was calculated as follows:

$$ \boldsymbol{M}\boldsymbol{T}\boldsymbol{S}=\frac{\boldsymbol{Sum}\ \boldsymbol{of}\ \boldsymbol{survival}\ \boldsymbol{time}\ \boldsymbol{of}\ \boldsymbol{all}\ \boldsymbol{mice}\ \boldsymbol{in}\ \boldsymbol{group}\ \left(\boldsymbol{days}\right)}{\boldsymbol{Total}\ \boldsymbol{number}\ \boldsymbol{of}\ \boldsymbol{mice}\ \boldsymbol{in}\ \boldsymbol{that}\ \boldsymbol{group}} $$

### Data analysis

Results of the study were expressed as a mean plus or minus standard error of mean (M ± SEM). Data were analyzed using Windows SPSS Version 16.0. One-way analysis of variance (ANOVA) followed by Tukey’s (post-hoc test) was used to determine statistical significance for comparison of parasitaemia, % suppression, body weight, PCV, rectal temperature and survival time among groups. The analysis was performed with 95% confidence interval and *P*-values less than 0.05 was considered to be statistically significant.

## Results

### Acute toxicity

The in vivo acute toxicity test indicated that both hydro-methanolic and aqueous extracts of leaves of *S. mitis* did not cause mortality and body weight reduction up to 2000 mg/kg oral doses within the first 24 h as well as for the subsequent 14 days. Gross physical and behavioral observation also revealed no visible signs of toxicity such as lacrimation, hair erection, and reduction in their motor and feeding activities.

### Extract yield

The leaves of *S. mitis* yielded a total of 56.8 g (18.8%) of dried hydro-methanolic crude extract and 43.3 g (14.4%) of dried aqueous crude extract.

### Phytochemical screening

Phytochemical screening of leaves of *Strychnos mitis* for the presence or absence of different secondary metabolites including alkaloids, tannins, saponins, flavonoids, terpenoids, steroids, phenols and glycosides gave the following results as shown in Table 1.

**Table 1:** Result of phytochemical screening of hydro-methanolic and aqueous extracts of leaves of *Strychnos mitis*

Phytochemical	Test results	
	Hydro-methanolic extract	Aqueous extract
Alkaloids	+	+
Tannins	+	+
Saponins	+	+
Flavonoids	_	_
Terpenoids	+	+
Steroids	+	+
Phenols	+	+
Glycosides	+	+

Note: + indicates the presence and − the absence of particular metabolites

### *In vivo* antiplasmodial activity of crude extracts on parasitaemia and survival time

The 4-day suppressive test results indicated that both hydro-methanolic and aqueous extract of the leaves of *S. mitis* had prominent antiplasmodial activity against chloroquine sensitive *P. berghei* infected Swiss albino mice (Table 2). The level of suppression of hydro-methanolic extract at concentrations of 200 mg/kg/day, 400 mg/kg/day and 600 mg/kg/day following the 4-day test was 36.56, 81.49 and 93.97%, respectively, and that of aqueous extract at concentrations of 200 mg/kg/day, 400 mg/kg/day and 600 mg/kg/day was 29.43, 74.86 and 95.5%, respectively.

**Table 2:** Effect of crude extract of the leaves of *S. mitis* on parasitaemia, percent suppression and survival time of *P. berghei* infected mice

Test substances	Dose (mg/kg)	% parasitaemia	%suppression	Survival time (day)
AE	200	31.65 ± 7.69	29.43 a^3^,c^3^,d^3^,e^3^	9.83 ± 1.32 a^2^,c^2^,d^3^,e^3^
	400	11.27 ± 4.05	74.86 a^3^,b^3^,d^2^,e^3^	12.33 ± 1.63 a^3^,b^2^,d^3^,e^3^
	600	2.01 ± 0.55	95.50 a^3^,b^3^,c^2^	17.50 ± 1.04 a^3^,b^2^,c^3^,e^3^
Vehicle	1 ml	44.85 ± 5.81	0.00	7.33 ± 0.81
CQ	25	0.00	100	27.50 ± 0.83
HE	200	28.45 ± 5.27	36.56a^3^,c^3^,d^3^,e^3^	10.83 ± 1.72a^2^,d^3^,e^3^
	400	8.30 ± 5.03	81.49a^3^,b^3^,e^2^	13.00 ± 2.00a^3^,e^3^,d^2^
	600	2.70 ± 1.10	93.97a^3^,b^3^	16.50 ± 1.04a^3^,b^3^,c^2^,e^3^
Vehicle	1 ml	44.85 ± 5.81	0.00	7.33 ± 0.81
CQ	25	0.00	100	27.50 ± 0.83

Data are expressed as mean ± SEM; *n* = 6; a = compared to distilled water (vehicle); b = compared to200 mg/kg; c = compared to 400 mg/kg; d = compared to 600 mg/kg; e = compared to chloroquine (25 mg/kg)

*AE* aqueous extract, *CQ* chloroquine, *HE* hydro-methanolic extract

^1^ = *p* < 0.05, ^2^ = *p* < 0.01, ^3^ = *p* < 0.001

Both hydro-methanolic and aqueous extracts of *S. mitis* showed statistically significant (*p* < 0.001) difference in reducing parasite load at all dose levels as compared to the negative control after the 4-day suppressive test. The respective two doses of both extracts (400 mg/kg and 600 mg/kg) demonstrated a statistically significant (*P* < 0.001) parasitaemia reduction as compared to the same extracts at a dose of 200 mg/kg. At higher dose (600 mg/kg), both aqueous and hydro-methanolic extracts showed higher percentage suppression, that is, 95.5 and 93.97% respectively, which is comparable to that of CQ (25 mg/kg) (100%).

There was significant difference in mean survival time (*p* < 0.01) between *P. berghei* infected mice treated with respective 200 mg/kg, 400 mg/kg and 600 mg/kg of hydro-methanolic and aqueous extracts of the leaves of *S. mitis* and infected mice in the negative control group following the 4-day suppressive test (Table 2).

### Effect of crude extracts on body weight

Two dose levels (400 mg/kg and 600 mg/kg) of the hydro-methanolic and aqueous extracts significantly protected parasite-induced weight reduction in infected mice as compared to those in the negative control groups (Table 3).

**Table 3:** Effect of crude extracts of *S. mitis* leaves on body weight of *P. berghei* infected mice

Test substance	Dose (mg/kg)	Body weight		% change
		D_0_ (g)	D_4_ (g)	
AE	200	30.33 ± 1.93	28.28 ± 3.82	−8.29 ± 9.97 c^1^,e^2^,d^2^
	400	29.83 ± 1.40	30.35 ± 1.35	1.65 ± 3.53 a^1^,b^1^
	600	29.40 ± 1.69	30.76 ± 1.73	4.41 ± 2.72 a^2^,b^2^
Vehicle	1 ml	29.26 ± 1.26	27.30 ± 1.10	−7.18 ± 1.52
CQ	25	29.11 ± 1.54	30.12 ± 2.03	3.21 ± 3.50
HE	200	31.33 ± 0.54	29.88 ± 2.58	−5.43 ± 8.30
	400	29.63 ± 1.45	30.16 ± 1.32	1.73 ± 3.442 a^1^
	600	30.41 ± 1.92	32.45 ± 2.23	6.22 ± 1.02 a^3^,b^2^
Vehicle	1 ml	29.26 ± 1.26	27.30 ± 1.10	−7.18 ± 1.52
CQ	25	29.11 ± 1.54	30.12 ± 2.03	3.21 ± 3.50

Data are expressed as mean ± SEM; *n* = 6; a = compared to distilled water (vehicle); b = compared to200 mg/kg; c = compared to 400 mg/kg; d = compared to600 mg/kg; e = compared to CQ 25 mg/kg

*D*_*0*_ pre-treatment value on day 0, *D*_*4*_ post-treatment value on day four, *AE* aqueous extract, *CQ* chloroquine, *HE* hydro-methanolic extract

^1^ = *p* < 0.05, ^2^ = *p* < 0.01, ^3^ = *p* < 0.001

### Effect of crude extracts of the leaves of *S. mitis* on packed cell volume and rectal temperature

**Fig. 1:**
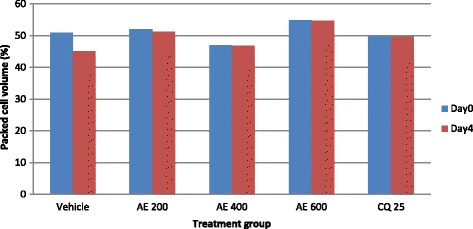
The effect of aqueous extract of leaves of *S. mitis* on packed cell volume of *P. berghei* infected mice on 4 day suppression test; data are mean ± SEM; *n* =6; CQ = chloroquine, AE = aqueous extract of *S. mitis*; numbers refer to doses in mg/kg/day

**Fig. 2:**
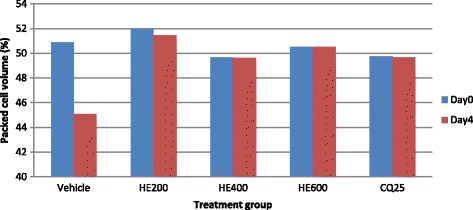
The effect of hydro-methanolic extract of leaves of *S. mitis* on packed cell volume of *P. berghei* infected mice on 4 day suppression test; data are mean ± SEM; *n* = 6; CQ = chloroquine, HE = hydro-methanolic extract of *S. mitis*; numbers refer to doses in mg/kg/day

The aqueous extract of the leaves of *S. mitis* significantly prevented reduction in PCV significantly at the higher two doses (400 mg/kg 600 mg/kg) as compared to the negative control with respective p values of <0.05 and <0.01 (Fig. 1). On the other hand, all the three doses of hydro-methanolic extract significantly (*p* < 0.01) prevented reduction in PCV as compared to the negative control (Fig. 2).

**Fig. 3:**
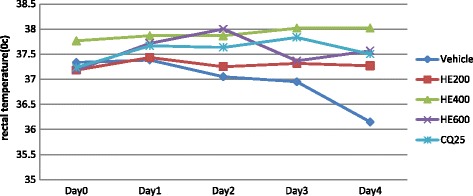
The effect of hydro-methanolic extract of *S. mitis* leaves on rectal temperature of *P. berghei* infected mice on 4-day suppression test; data are mean ± SEM; *n* = 6; CQ = chloroquine, AE = aqueous extract of *S. mitis*; numbers refer to doses in mg/kg/day

**Fig. 4:**
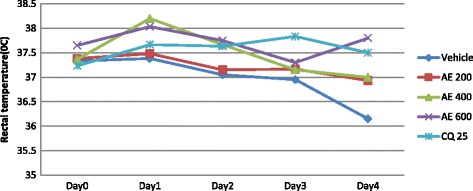
The effect of aqueous extract of *S. mitis* leaves on rectal temperature of *P. berghei* infected mice on 4-day suppression test; data are mean ± SEM; *n* = 6; CQ = chloroquine, AE = aqueous extract of *S. mitis*; numbers refer to doses in mg/kg/day

All the three doses of the hydro-methanolic extract of the plant significantly prevented reduction in rectal temperature as compared to the negative control: 600 mg/kg with p value <0.001 and 200 mg/kg and 400 mg/kg with p values < 0.01. At the dose level of 600 mg/kg, the effect of the extract on rectal temperature is comparable to CQ (25 mg/kg) (Fig. 3). Whereas, the aqueous extract of the plant significantly (*p* < 0.05) prevented reduction in rectal temperature only at the dose of 600 mg/kg as compared to the negative control (Fig. 4).

### Effect of fractions of leaves of *S. mitis* on parasitaemia, percent suppression and survival time

At all dose levels evaluated, all the fractions showed statistically significant (*p* < 0.001) difference in reducing parasite load in mice as compared to the negative control after 4-day suppressive test. The percent suppressions of the solvent fractions n-hexane, chloroform and aqueous were 42.85%), 39.72 and 34.47%, respectively. Survival dates were significantly prolonged by all dose levels as compared to negative control (Table 4).

**Table 4:** Effect of fractions of leaves of *S. mitis* on percent parasitaemia, percent suppression and survival time on *P. berghei* infected mice

Test substances	Dose (mg/kg)	% parasitaemia	% suppression	Survival time (day)
NF	100	28.68 ± 6.31	42.11 a^3^,e^3^	11.33 ± 1.21a^3^,e^3^
	200	28.31 ± 0.89	42.85 a^3^,e^3^	11.50 ± 1.76 a^3^.e^3^
	400	32.81 ± 4.38	33.77 a^3^,e^3^	11.00 ± 1.54 a^3^,e^3^
CF	100	36.25 ± 2.14	26.84 a^3^,d^3^.e^3^	10.50 ± 0.54 a^3^,e^3^
	200	33.83 ± 3.19	31.71a^3^,d^1^,e^3^	10.83 ± 0.75 a^3^,e^3^
	400	29.86 ± 2.30	39.72 a^3^,b^3^,c^1^,e^3^	11.33 ± 1.03 a^3^,e^3^
AF	100	37.26 ± 1.84	24.78 a^3^,d^3^,e^3^	10.16 ± 1.47 a^2^,e^3^
	200	36.10 ± 2.52	27.14a^3^,d^2^,e^3^	10.66 ± 1.36 a^3^,e^3^
	400	32.46 ± 2.18	34.47 a^3^,b^3^,c^2^,e^3^	11.16 ± 0.75 a^3^,e^3^
Vehicle	1 ml	49.55 ± 2.53	0.00	7.33 ± 1.03
CQ	25	0.00	100	27.83 ± 0.75

Data are expressed as mean ± SEM; *n* = 6; a = compared to distilled water (vehicle); b = compared to100 mg/kg; c = compared to 200; d = compared to400 mg/kg; e = compared to chloroquine 25 mg/kg

*CQ* chloroquine, *NF* n-hexane fraction of crude hydro-methanolic extract, *CF* chloroform fraction of crude hydro-methanolic extract, *AF* aqueous fraction of Crude hydro-methanolic extract

^1^ = *p* < 0.05, ^2^ = *p* < 0.01, ^3^ = *p* = <0.001

### Effect of fractions of leaves of *S. mitis* on body weight

All tested doses of the n-hexane fraction and the highest two doses (200 mg/kg and 400 mg/kg) of chloroform and aqueous fractions of *S. mitis* significantly protected the mice from body weight loss as compared to negative control after 4-day suppressive test (Table 5).

**Table 5:** Effect of fraction of *S. mitis* leaves on body weight of *P. berghei* infected mice

Test substances	Dose (mg/kg)	Body weight		% Change
		D0 (g)	D4 (g)	
NF	100	29.45 ± 1.46	30.48 ± 2.12	3.26 ± 2.68 a^2^
	200	29.40 ± 1.65	30.36 ± 2.47	2.94 ± 4.73 a^2^
	400	30.25 ± 1.63	30.86 ± 2.34	1.73 ± 5.74 a^1^
CF	100	29.68 ± 1.55	29.28 ± 1.56	−1.40 ± 2.86
	200	29.75 ± 1.42	30.40 ± 2.37	1.89 ± 4.47 a^2^
	400	30.46 ± 1.70	31.11 ± 2.35	1.89 ± 4.02 a^2^
AF	100	29.43 ± 2.06	28.93 ± 2.02	−1.79 ± 4.07
	200	29.41 ± 1.79	29.75 ± 2.26	1.00 ± 2.75 a^1^
	400	30.76 ± 0.91	31.35 ± 1.28	1.70 ± 5.20 a^2^
Vehicle	1 ml	30.51 ± 1.78	28.20 ± 1.59	−8.39 ± 7.04
CQ	25	29.23 ± 2.00	30.74 ± 2.53	4.79 ± 2.10 a^3^

Data are expressed as mean ± SEM; *n* = 6; a = compared to distilled water (vehicle), b = compared to100 mg/kg, c = compared to 200, d = compared to400 mg/kg, e = compared to chloroquine 25 mg/kg

*D0* pre-treatment value on day 0, *D4* post-treatment value on day four, *CQ* chloroquine, *NF* n-hexane fraction of crude hydro-methanolic extract, *CF* chloroform fraction of crude hydro-alcoholic extract, *AF* aqueous fraction of crude hydro-methanolic extract

^1^ = *p* < 0.05, ^2^ = *p* < 0.01, ^3^ = *p* < 0.001

### Effect of fractions of leaves of *S. mitis* on packed cell volume and rectal temperature

**Fig. 5:**
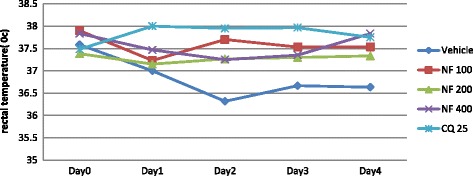
The effect of n-hexane fraction of leaves of *S. mitis* on rectal temperature of *P. berghei* infected mice on 4-day suppression test. Data are mean ± SEM; *n* = 6; CQ = chloroquine, NF = hexane fraction; numbers refer to doses in mg/kg/day

**Fig. 6:**
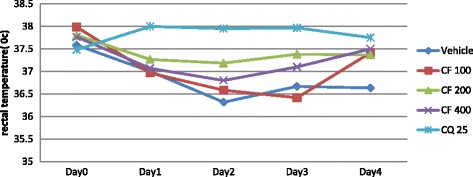
The effect of chloroform fraction of leaves of *S. mitis* on rectal temperature of *P. berghei* infected mice on 4-day suppression test. Data are mean ± SEM; *n* = 6; CQ = chloroquine, CF = chloroform fraction; numbers refer to doses in mg/kg/day

**Fig. 7:**
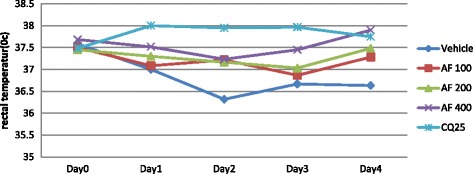
The effect of aqueous fraction of leaves of *S. mitis* on rectal temperature of *P. berghei* infected mice on 4-day suppression test. Data are mean ± SEM; *n* = 6; CQ = chloroquine, AF = aqueous fraction; numbers refer to doses in mg/kg/day

None of the doses of the fractions of *S. mitis* significantly improved body temperature of *P. berghei* infected mice as compared to the negative control as indicated in Figs. 5, 6 and 7.

**Fig. 8:**
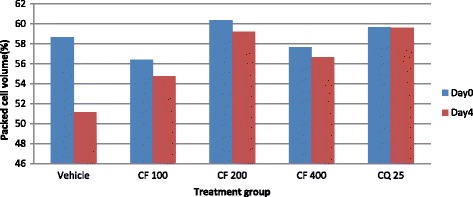
The effect of chloroform fraction of leaves of *S. mitis* on packed cell volume of *P. berghei* infected mice on 4-day suppression test. Data are mean ± SEM; *n* = 6; CQ = chloroquine, CF = chloroform fraction; numbers refer to doses in mg/kg/day

**Fig. 9:**
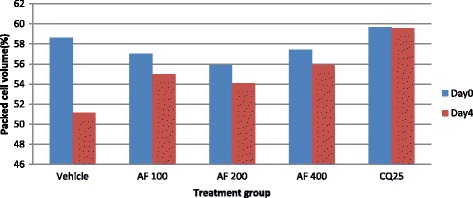
The effect of aqueous fraction of leaves of *S. mitis* on packed cell volume of *P. berghei* infected mice on 4-day suppression test. Data are mean ± SEM; *n* = 6; CQ = chloroquine, AF = aqueous fraction; numbers refer to doses in mg/kg/day

**Fig. 10:**
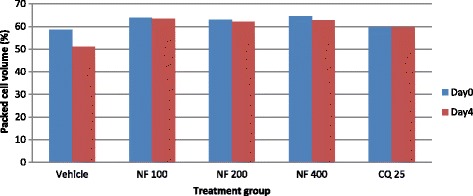
The effect of n-hexane fraction of leaves of *S. mitis* on packed cell volume of *P. berghei* infected mice on 4 day suppression test. Data are mean ± SEM; *n* = 6; CQ = chloroquine, NF = hexane fraction; numbers refer to doses in mg/kg/day

All dose levels of chloroform fraction of *S. mitis* significantly (*p* < 0.01) prevented reduction in PCV as compared to the negative control (Fig. 8). Similarly, all dose levels of aqueous fraction demonstrated significantly prevented PCV reduction (400 mg/kg, *p* < 0.01; 20 mg/kg, *p* < 0.01; 100 mg/kg, *p* < 0.05) as compared to the negative control (Fig. 9). The n-hexane fraction also significantly prevented PCV reduction (400 mg/kg, *p* < 0.05; 100 mg/kg, *p* < 0.01; 200 mg/kg, *p* < 0.01) as compared to the negative control (Fig. 10).

## Discussion

The observation that no death caused by an oral dose of 2000 mg/kg body weight of the hydro-methanolic and aqueous extracts of the leaves of *S. mitis* could imply the safety of the plant to be used in the treatment of malaria as also suggested in Akele [31] and Murithi et al. [32]. The acute toxicity result of the present study suggested that the oral medial lethal dose (LD_50_) of the extract could be greater than 2000 mg/kg body weight as per OECD guideline No 425 [27]. The experimental determination of lack of acute toxicity at the extract dose of up to 2000 mg/kg body weight of mice may justify the use of this plant for malaria treatment.

In vivo antiplasmodial activity can be classified as moderate, good and very good if an extract displayed a respective percent parasite suppression equal to or greater than 50% at doses of 500, 250 and 100 mg/kg body weight per day [33, 34]. Based on this classification, the crude extracts of *S. mitis* are considered to have exhibited good antiplasmodial activity, with dose dependent inhibition against *P. berghei* infection in mice.

Analysis of test results indicated significant parasitaemia suppression by all the doses of hydro-methanolic and aqueous extracts of *S. mitis* as compared to the negative control after the 4-day suppression test. The parasite suppression exhibited by these extracts is comparable to results of former studies conducted on methanol extract of the leaves of *Aloe debrana* [11], crude extract of *Croton macrostachyus* Del. [35] and hexane extract of *Ficus thonningii* [36]. Different studies [15, 17–20] revealed the antiplasmodial activity of *Strychnos gossweileri*, *Strychnos icaja*, *Strychnos mellodora*, *Strychnos spinosa*, *Strychnos usambarensis* and *Strychnos variabilis*, all close relatives of *S. mitis*.

The n-hexane and chloroform fractions of *S. mitis* were found to demonstrate higher percentage of parasitaemia as compared to the aqueous fraction of the plant possibly suggesting the better availability of active ingredients in the former two fractions.

All the crude extracts and fractions of the plant prolonged the mean survival time of the experimental mice indicating that the plant suppressed *P. berghei* and reduced the overall pathologic effect of the parasite on the mice. However, neither the extracts nor the standard drug cured the infection. This could be due to recrudescence of *P. berghei* parasites after apparent cure. Similar result on mean survival time of mice was reported by Bantie et al. [35] and Mengiste et al. [10] in studies conducted on *Croton macrostachyus* and *Dodonaea viscosa* subsp. *angustifolia*, respectively. The longest survival time of mice as a result of the administration of the highest dose (600 mg/kg) of hydro-methanolic and aqueous extracts could be linked to the presence of active secondary metabolites in sufficient concentration in that dose. The phytochemical screening of hydro-methanolic and aqueous extract of *S. mitis* indicated the presence of alkaloids, anthraquinones, terpenoids, glycosides, saponins, tannins and phenolic compounds. As explained by Dharani et al. [37], common antimalarial plants used to treat malaria in traditional medicine contain secondary metabolites, such as alkaloids, terpenoids, coumarins, flavonoids, chalcones, quinines and xanthones. Alkaloids, terpenoids and tannins detected in *S. mitis* have been implicated for their antiplasmodial activity in previous study [38, 39]. Quinine, one of the most important and oldest antimalarial drugs, belongs to the class of alkaloids [40]. Phenolic compounds present in *S. mitis* could also possibly be responsible for the antiplasmodial activity as these metabolites have been proved to possess potential antimalarial effect in other studies [41, 42]. Phenolic compounds detected in the leaf extract of *S. mitis* were indicated to have antioxidant properties (free radical inhibitors or scavengers) in a study conducted by Adamu et al. [43] and that may contribute to the antiplasmodial activity of the plant. Antioxidative activity inhibits heme polymerization as heme needs to be oxidized before polymerization; unpolymerised heme is very toxic to the parasite [44].

Anemia, body weight loss and body temperature reduction are the general features of malaria-infected mice [45]. Thus antimalarial agents are expected to prevent body weight loss in infected mice due to rise in parasitaemia. The crude extracts (aqueous and hydro-methanolic) and fractions (chloroform and aqueous) of leaves of *S. mitis* significantly prevented weight loss at their higher two doses in a dose dependent manner. Whereas, n-hexane fraction of the plant significantly prevented weight loss at all dose levels in a dose independent manner suggesting the possibility of localization of appetite-suppressing components, and nutrients and other immunomodulatory substances even at the lower dose of this fraction. Comparable effects in preventing weight loss were also reported in studies conducted on hydro-alcoholic extract of A*sparagus africanus* obtained [13, 35].

A decrease in the metabolic rate of infected mice occurs before death and is accompanied by a corresponding decrease in internal body temperature [10]. All the doses of hydro-methanolic extract and the highest dose of aqueous extract demonstrated protective effect against temperature reduction, likely suggesting the presence of constituents in the extracts responsible for such effect. A study by Sanmugapriya and Venkataraman [21] revealed the antipyretic effect of the seeds of *Strychnos potatorum*, a close relative of *S. mitis*. The effects on rectal temperature by *S. mitis* is comparable to that reported in previous study conducted on crude extract and chloroform fraction of *Croton macrostachyus* [35]. Unlike the crude extracts of *S. mitis*, all fractions of the plant failed to significantly prevent parasite induced rectal temperature reduction as compared to the negative control. This could be attributed to the effect by the fractions themselves as they may have hypothermic effect on the treated mice.

Both the crude extracts and fractions of the leaves of *S. mitis* significantly prevented PCV reduction in a dose dependent manner as compared to the negative control. Comparable effects on PCV were reported by previous studies conducted on *Dodonaea viscosa* subsp. *angustifolia* [10] and *Croton macrostachyus* [35].

## Conclusion

The present study indicated promising in vivo antiplasmodial effect of the crude extracts and solvent fractions of *S. mitis*. The extracts were also found to be safe at the maximum dose of 2000 mg/kg. The antimalarial effect of the solvent fractions of the hydro-methanolic extract was revealed to be less as compared to that by the crude hydro-methanolic extract. The n-hexane fraction had protected body weight loss at all dose levels and displayed greater parasite suppression as compared to the other fractions. Therefore, the extracts and fractions of hydro-methanolic extract could potentially be used as a new source for the development of new plant-based antimalarial agent. Moreover, the data could be used as additional evidence to uphold claims by the Ethiopian traditional medicine practitioners on the effect of traditionally used plants to manage malaria.
